# Inositols in Insulin Signaling and Glucose Metabolism

**DOI:** 10.1155/2018/1968450

**Published:** 2018-11-25

**Authors:** Arturo Bevilacqua, Mariano Bizzarri

**Affiliations:** ^1^Department of Dynamic and Clinical Psychology, Sapienza University of Rome, via dei Marsi 78, 00185 Rome, Italy; ^2^Center for Research in Neurobiology “Daniel Bovet” (CRiN), Sapienza University of Rome, 00185 Rome, Italy; ^3^Department of Experimental Medicine, Systems Biology Group Lab, Sapienza University of Rome, via A. Scarpa 16, 00161 Rome, Italy

## Abstract

In the past decades, both the importance of inositol for human health and the complex interaction between glucose and inositol have been the subject of increasing consideration. Glucose has been shown to interfere with cellular transmembrane transport of inositol, inhibiting, among others, its intestinal absorption. Moreover, intracellular glucose is required for de novo biosynthesis of inositol through the inositol-3-phosphate synthase 1 pathway, while a few glucose-related metabolites, like sorbitol, reduce intracellular levels of inositol. Furthermore, inositol, via its major isomers *myo*-inositol and D-*chiro*-inositol, and probably some of its phosphate intermediate metabolites and correlated enzymes (like inositol hexakisphosphate kinase) participate in both insulin signaling and glucose metabolism by influencing distinct pathways. Indeed, clinical data support the beneficial effects exerted by inositol by reducing glycaemia levels and hyperinsulinemia and buffering negative effects of sustained insulin stimulation upon the adipose tissue and the endocrine system. Due to these multiple effects, myoIns has become a reliable treatment option, as opposed to hormonal stimulation, for insulin-resistant PCOS patients.

## 1. Inositol and Insulin Signaling System

It is currently well known that diets providing high amounts of fibers have a protective role in the management (and prevention) of chronic diseases—namely those characterized by deregulated glucose and insulin metabolism [1, 2]. While consumption of highly refined diets is frequently associated with increased risk for diabetes and cancer [3], the effect of high-fiber diets is usually ascribed to the presence of whole-grain cereal products and of a plethora of phytochemicals, which display an astonishing range of different biochemical-pharmacological effects, well documented in *in vitro* and *in vivo* studies [4]. Among the aforementioned constituents, inositol appears to occupy a prominent position. Indeed, several investigations have demonstrated that only fibers with high content in phytic acid or phytate (inositol hexakisphosphate, InsP6) and its derivative *myo*-inositol (myoIns) show negative correlation with colon cancer and diabetes, indicating that inositol-derived compounds can suppress colon carcinogenesis [5] or improve glucose metabolism [6].

Indeed, several alterations in inositol metabolism have been observed in insulin-resistant and diabetic patients ([7], reviewed in [8]). Namely, the decreased availability of inositol or inositol phosphoglycans (IPGs) can be primarily ascribed to increased urinary loss of myoIns in both animals and humans with insulin resistance. This effect is mostly due to the glucose-mediated inhibition of myoIns reabsorption by the kidney. Reduction in myoIns availability has a direct negative impact on the levels of its D-*chiro*-inositol (DCIns) isomer. In turn, plasma and intracellular depletion of myoIns and its isomers or IPG metabolites is likely to worsen insulin resistance. In fact, IPGs and probably myoIns itself are involved in the insulin signaling machinery. Conversely, altered profiles of plasma/urinary levels of inositol and its metabolites/isomers, including DCIns, have been observed in diabetes, insulin resistance, or metabolic syndrome.

These findings boosted investigation on the involvement of inositol in different physiological conditions, comprising fertility [9], oogenesis [10], embryogenesis [11, 12] regenerative processes [13], and fat metabolism [14].

It is currently acknowledged that some of the effects ascribed to inositol can be explained by the ability of this molecule or its metabolites to modulate glucose metabolism, by transducing insulin effects downstream of insulin-receptor (IR) coupling. Yet, the complex interplay among insulin, glucose metabolism and inositol-related molecules is still poorly understood and only a few surveys have been hitherto performed to address such issue.

## 2. Inositol Availability and Dietary Supply

Inositol is chemically identified as hexahydroxycyclohexane and consists of a family of 9 stereoisomers. MyoIns is the most widely distributed representative of this family in nature, including animals and mammals [15]. A normal diet provides inositols, mainly present in cereals and legumes, as myoIns, for the most part, phosphatidylinositol (PI) and InsP6 [16]. Inositol has been shown to be essential for cell growth and has been considered for a long time as a component of the vitamin B family [17, 18]. However, we now know that the human body (especially the liver and brain) [19] can produce up to 4 g/day inositol, and that assumption is no longer recognized.

Indirect estimates of the availability of myoIns in human diet, based on the consumption of phytate-rich food, reveal daily intakes not exceeding 500–700 mg/day for western countries, with higher consumption rates in Africa and Asia. In Italy, for example, available estimates of the mean value of phytic acid intake range from 219 to 293 mg/day [20, 21]. In the USA and Canada, average phytic acid intake is of 538 mg/day in adults, with relevant differences between males and females (608 mg vs 512 mg/day, respectively) [22], and of 170–390 mg/day in children [23].

## 3. Glucose and Inositol Biosynthesis

As anticipated, myoIns is actively synthetized by living cells. We do not know if this biosynthetic activity makes our body independent of dietary inositol supply, but an idea of how relevant inositol biosynthesis can be in some tissues comes from observations on the brain, which produces myoIns at concentrations reaching 10- to15-fold the values measured in circulating blood, with only limited “extracting” ability [24].

The cellular precursor of myoIns is glucose-6-phosphate (G6P), which is isomerized to inositol-3-phosphate (Ins3P) by D-3-*myo*-inositol-phosphate synthase (inositol synthase, Ino1 or MIPS1), an enzyme encoded by the evolutionarily conserved Ino1 and Isyna1 genes in yeast and mammals, respectively [24]. Inositol-3-phosphate is then dephosphorylated to free myoIns by inositol monophosphatase-1 (IMPA-1 or IMPase) [25]. Free inositol may also be obtained by recycling inositol-1,4,5-trisphosphate (InsP3) and inositol-1,4-bisphosphate (InsP2).

MyoIns biosynthesis varies among tissues depending on changing functional requirements. In yeast, Ino1 and IMPA-1 are induced by low inositol levels [26] and downregulated by high inositol levels [27]. Both Ino1 and Isyna1 can be epigenetically modified [28] and mammalian Isyna1 exhibits gender- and tissue-specific DNA methylation patterns [29] that modulate inositol biosynthesis.

In mammalian cells, however, intracellular myoIns availability seems ineffective in controlling Isyna1 activity [30], the expression of which is downregulated by inositol pyrophosphates (IP7), produced by inositol hexakisphosphate kinase (IP6K1) [31]. IP7 has been found to inhibit transcription of Isyna1 by increasing its methylation levels [32]. A key role in this pathway is played by phosphatidic acid (PA), which increases nuclear translocation of IP6K1, thus finally leading to decreased myoIns content. PA increases when higher energy demand imposes high rates of glucose catabolism [33], being synthesized from two glycolytic intermediates—dihydroxyacetone phosphate and glycerol-3-phosphate [34]. Furthermore, high glucose levels indirectly increase the activity of phospholipase D (PLD), a key enzyme for PA synthesis [35].

In mammalian cells, a positive regulator of Isyna1 transcription is glycogen synthase kinase 3 (GSK3), since loss of GSK3 activity causes myoIns depletion [36]. Furthermore, synthesis of inositol is positively regulated by Mck1, a GSK3 homolog required for normal activity of MIPS1, the rate-limiting enzyme in inositol synthesis [37].

## 4. Influence of Glucose on Inositol Uptake and Metabolism

Long ago, a negative association observed between phytic acid intake and the glycemic index of cereals and legumes consumed by humans led to a hypothesis of a correlation among inositol, phytic acid, and glucose metabolism [38]. This was supported by further studies, which revealed that removal of phytate from bean flour increases its glycemic index [39] and that myoIns significantly inhibits duodenal glucose absorption and reduces blood glucose rise, suggesting the existence of a competitive affinity for the same transporter system [40].

It now appears that a complex relationship between glucose and myoIns does exist, and glucose can interfere with myoIns metabolism at different levels.

In the first place, glucose significantly counteracts cellular uptake of inositol. Inositol is transported intracellularly by sodium ion-coupled transporters (SMIT1 and SMIT2) [41], which are posttranslationally regulated through phosphorylation by both protein kinase A and protein kinase C [42], and a proton-coupled transporter (HMIT1) [43].

In hepatocytes, inhibitors of the main sodium-glucose transporters (SGLT) 1/2 prevent both glucose and inositol uptake [44], suggesting that the two molecules share the transporter system(s). Inositol uptake is also decreased by glucose. MyoIns depletion in nervous tissues induced by hyperglycemia depends on competitive inhibition of sodium ion-coupled myoIns transporters [45]. Inositol uptake in cultured cells is also significantly inhibited by 20 mM glucose [46].

Secondly, glucose may induce myoIns depletion by activation of the glucose-sorbitol pathway, in which glucose is first converted to sorbitol by aldose reductase and then to fructose by sorbitol dehydrogenase, as observed in diabetic patients [47]. The increased production of sorbitol produces a harmful rise in intracellular osmolarity, an event counteracted by inhibition of the uptake of other relevant osmolytes, including inositol, via downregulated expression of their carriers [48]. Inhibiting aldose reductase in cultured cells restores myoIns levels counteracting the depleting effect of sorbitol [49].

Glucose-dependent depletion of normal levels of intracellular inositol is further confirmed by observations obtained on specific tissues, especially in those susceptible of developing diabetes complications, of hyperglycemic patients [50]. In addition, both hyperglycemia and insulin resistance have been found to modify the relative ratio in which different inositol isomers, especially myoIns and DCIns (approximately 100 : 1), are present in these tissues [51]. Changes in the ratio of plasma and urinary myoIns/DCIns levels are so tightly linked to insulin abnormalities to be considered an early marker of hyperglycemia and insulin resistance [7]. This worsens insulin resistance and diabetes complications, impairs cellular redox and free radical defenses, and increases oxidative glycation stress [52, 53]. Under these circumstances, myoIns supplementation reduces several diabetes symptoms and metabolic markers of different pathologies [54].

## 5. Inositol Mechanisms of Action

A role for inositol and inositol-related metabolites in insulin signaling emerged from the observation that, under insulin stimulation and activation of a phosphatidyl-inositol-specific phospholipase C, liver plasma membrane releases inositol phosphoglycans (IPG) containing either myoIns (IPG-A) or DCIns (IPG-P) [55]. While IPG-P (inositol phosphoglycan-phosphatase stimulator) promotes the activation of pyruvate dehydrogenase (PDH) phosphatases (PDHP) and PDH [56], IPG-A (inositol phosphoglycan-AMP kinase inhibitor) inhibits both protein kinase A and adenylyl cyclase (AC) [57, 58].

Both types of IPGs have been shown to exert an insulin-mimetic activity, acting as second messengers downstream of insulin receptors [56]. When administered to normal or diabetic rats, they reduce hyperglycemia in a dose-dependent fashion and promote muscular glycogenesis [59]. Insulin stimulates glycosyl-phosphatidylinositol (GPI) hydrolysis through direct activation of phospholipase C (PLC) and PLD, with production of water-soluble IPSs [60, 61]. Inositol phosphoglycans also inhibit AC and protein kinase A with antilipolytic and lipogenic effects [62].

Upon insulin receptor activation, IPGs are released outside the cell and reimported by an ATP-dependent inositol glycan transporter [63], activating cytosolic phosphoprotein phosphatase 2C-*α* (PP2C*α*) and mitochondrial PDH. This enhances oxidative glucose metabolism along the tricarboxylic acid cycle. Activated PP2C*α* stimulates glycogen synthase (GS) both directly and indirectly, through the phosphatidylinositide 3-kinase (PI3K)/Akt pathway [64]. Activation of Akt inactivates glycogen synthase kinase-3 (GSK-3), enhancing GS activity, and increases GLUT-4 translocation and glucose uptake. Finally, as observed in the rat, IPG-P inhibits the glucose-stimulated release of insulin from pancreatic *β*-cells, suggesting the existence of a negative feedback mechanism in this pathway [65]. On these bases, IPGs are supposed to exert a general antidiabetic function.

The proper ratio at which IPG-A and IPG-P display the most beneficial effects on insulin/glucose homeostasis is still unclear, besides the fact that their contemporary presence appears mandatory.

## 6. Hypotheses for Future Research

We would hypothesize that DCIns containing IPG is preferentially synthetized during metabolic stress, in response to increased insulin release. Indeed, upon insulin stimulation, myoIns is converted by a specific epimerase into its DCIns isomer. Epimerase activity is strictly dependent on insulin, and shows a tissue-dependent modulation [66]. Inositol isomerization occurs at the phospholipid level where (^3^H)-myoIns phospholipid are converted into (^3^H)-DCIns as soon as 15 minutes after insulin stimulation [67]. However, we cannot discard the possibility that epimerization also occurs at the free inositol level, but with the subsequent rapid incorporation of DCIns into phospholipids, as observed both in vitro and in vivo [68, 69]. Conversely, myoIns epimerization is severely impaired when insulin-sensitive tissues (muscle, fat, and liver) become insulin resistant, and a reduced DCIns/myoIns ratio has been suggested to represent a measure of insulin resistance [70]. Accordingly, reduced DCIns levels are typical of urine and muscle tissue of type 2 diabetic patients [71, 72]. Additionally, insulin resistance in both type 2 diabetic subjects with impaired glucose tolerance and healthy controls appears to be related linearly with urinary deficiency of DCIns [73]. Yet, insulin stimulates IPG-P release in normal subjects but not in insulin-resistant women with polycystic ovary syndrome (PCOS) [74]. These observations may be the result of a deficiency in membrane-bound IPG and/or insulin resistance with reduction of epimerase-dependent conversion of myoIns into DCIns.

Intriguingly, in both insulin-resistant animals and humans, the decreased availability of inositol or IPGs is associated with the increased urinary loss of myoIns [75], probably depending on glucose-mediated inhibition of renal myoIns reabsorption [76]. Ultimately, a shortage of myoIns impacts negatively on DCIns levels [77], worsening insulin resistance. Indeed, plasma and tissue levels of DCIns and IPGs are decreased in PCOS patients with insulin resistance and type 2 diabetic patients [76]. However, treatment with myoIns has not been found to lower insulin and glycaemia in all patients with metabolic syndrome [78], strongly suggesting that sensitivity to inositol supplementation of insulin-resistant subjects may depend on still unknown additional factors.

This picture is further complicated by an intriguing paradox that emerged in recent studies on PCOS patients displaying insulin resistance. While insulin-resistant tissues do suffer from a general paucity of DCIns due to the reduced epimerization of myoIns, organs and tissues that retain their insulin sensitivity, like the ovary [79], display increased levels of DCIns and reduced levels of myoIns, due to the enhanced epimerization of myoIns [80]. The impairment of ovarian function in PCOS patients has been specifically associated with the increase in the DCIns/myoIns ratio within the ovary [81].

Noticeably, the role of inositol-containing phosphoglycans in insulin regulation has been recently questioned by evidencing that several synthetic IPGs, opposite to natural IPGs, are deprived of insulin-mimetic activity [82]. Since no convincing explanation of such enigma has been proposed, it can be hypothesized that synthetic IPGs lack some critical cofactors required for the insulin-mimetic activity.

Other complications of insulin-inositol relationships come from the demonstration that in response to insulin stimulation of normal cells, myoIns may directly promote activation of insulin receptor substrate (IRS) and Akt [83]. In addition, myoIns and other inositol derivatives enhance GLUT-4 translocation through the cell membrane independently of insulin stimulation [84] or glucose uptake [85].

Overactivation of IP6K1, usually triggered by insulin stimulation, has been recently found to promote synthesis of IP7, which inhibits Akt activity by preventing its interaction with PI3K [86], thus reducing insulin sensitivity and protein synthesis via the GSK3*β* and mTOR signaling pathways, which are both associated with insulin resistance and weight gain [87]. On the contrary, IP6K1 knockout mice manifest insulin sensitivity and are resistant to obesity elicited by high-fat diet or aging.

To properly address such issues, we argue that a preliminary effort should be made by performing a thorough investigation of intracellular myoIns metabolism. Indeed, once uptaken by cells, myoIns undergoes several transformations into key active metabolites and macromolecular complexes, such as phosphoinositides and phospholipids. To date, despite widespread studies carried out to identify inositol-based effects on biological pathways, no comprehensive metabolomic analysis has been performed so far. Only an integrated metabolomic-genomic study would indeed provide the basic information required to identify the cellular fate of myoIns and its gene/protein targets.

## 7. Conclusions

Available data suggest that inositol, through its isomers (myoIns and DCIns) and probably some of its phosphate intermediate metabolites and correlated enzymes (like IP6K1), participates in insulin signaling and glucose metabolism, by influencing distinct pathways. Indeed, some preliminary clinical reports provided compelling evidence supporting the beneficial effects obtained with inositol in insulin-resistant patients. Inositol reduces glycemia levels and hyperinsulinemia, while buffering negative effects of sustained insulin stimulation upon the adipose tissue and the endocrine system [14, 88]. As such, myoIns has become a reliable treatment option for insulin-resistant PCOS patients [89, 90].

However, we are just unveiling the complex interplay between inositol(s) and insulin-related pathways. Namely, it is still unclear if insulin resistance depends (at least in part) on a reduced content of membrane-bound IPGs, on a primary defect in IPG release, or if impaired epimerase activation downstream of insulin stimulation would ultimately lead to reduced DCIns intracellular availability.

Anyhow, compelling evidence suggests that reduced DCIns levels are associated with impaired insulin transduction in insulin-sensitive tissues, where insulin-sensitive epimerase is unable to convert myoIns into DCIns. On the contrary, in insulin-insensitive tissues (like the ovary and placenta), insulin overstimulation induces an anomalous increased conversion of myoIns into DCIns [91]. The increased availability of DCIns in these tissues leads to several biological dysfunctions. Indeed, experimental evidence suggests that high DCIns levels in ovary and placenta cells can trigger paradoxical effects on insulin signaling. In preeclampsia-suffering women, an abnormal intracellular amount of DCIns within the placenta promotes a specific phosphorylation of insulin receptor substrate 1 (IRS-1) on serine^312^ and leads to inhibition of the PI3K/Akt pathway [92]. Akt inhibition worsens insulin resistance and hampers several pathways downstream of insulin stimulation, as observed in PCOS women whose treatment with high doses of DCIns was shown to be detrimental [93]. Interestingly, these results are confirmed by analysis of an experimental PCOS mouse model in which administration of myoIns and DCIns at ratios of 5 : 1 and 20 : 1 is detrimental for ovarian morphology and function, whereas their administration at ratios of 40 : 1 and at a lesser extent 80 : 1 rapidly restores physiological ovarian parameters [94].

To sum up, the interplay among the different isomers of inositol and their glycan derivatives (IPG-A and IPG-P) is still unclear and in depth studies are warranted to fully appreciate the contribution of inositol and inositol glycans in insulin signaling.

## Abbreviations

AC: Adenylyl cyclase
DCIns: D-*chiro*-Inositol
G6P: Glucose-6-phosphate
GLUT: Glucose transporter
GPI: Glycosyl-phosphatidylinositol
GSK: Glycogen synthase kinase
HMIT: H+/*myo*-inositol cotransporter
IMPA: IMPase, inositol monophosphatase
Ino1: Inositol synthase
Ins3P: Inositol-3-phosphate
InsP2: Inositol-1,4-bisphosphate
InsP3: Inositol-1,4,5-trisphosphate
InsP6: Inositol hexakisphosphate
IP6K1: Inositol hexakisphosphate kinase
IP7: Inositol pyrophosphates
IPG-A: myoIns phosphoglycans
IPG-P: DCIns phosphoglycans
IPGs: Inositol phosphoglycans
IR: Insulin receptor
IRS: Insulin receptor substrate
Isyna: Inositol-3-phosphate synthase
MIPS: *myo*-Inositol-phosphate synthase
mTOR: Mammalian target of rapamycin
myoIns: *myo*-Inositol
PA: Phosphatidic acid
PCOS: Polycystic ovary syndrome
PDH: Pyruvate dehydrogenase
PDHP: Pyruvate dehydrogenase phosphatase
PI3K: Phosphatidylinositide 3-kinase
PLC: Phospholipase C
PLD: Phospholipase D
PP2C*α*: Cytosolic phosphoprotein phosphatase 2C-*α*
SGLT: Sodium-glucose transporter
SMIT: Sodium-*myo*-inositol cotransporter.
